# Thermal Stress Induced Aggregation of Aquaporin 0 (AQP0) and Protection by α-Crystallin *via* Its Chaperone Function

**DOI:** 10.1371/journal.pone.0080404

**Published:** 2013-11-27

**Authors:** Satyanarayana Swamy-Mruthinti, Volety Srinivas, John E. Hansen, Ch Mohan Rao

**Affiliations:** 1 University of West Georgia, Carrollton, Georgia, United States of America; 2 Center for Cellular and Molecular Biology, Hyderabad, India; University of Alabama at Birmingham, United States of America

## Abstract

Aquaporin 0 (AQP0) formerly known as membrane intrinsic protein (MIP), is expressed exclusively in the lens during terminal differentiation of fiber cells. AQP0 plays an important role not only in the regulation of water content but also in cell-to-cell adhesion of the lens fiber cells. We have investigated the thermal stress-induced structural alterations of detergent (octyl glucoside)-solubilized calf lens AQP0. The results show an increase in the amount of AQP0 that aggregated as the temperature increased from 40°C to 65°C. α-Crystallin, molecular chaperone abundantly present in the eye lens, completely prevented the AQP0 aggregation at a 1∶1 (weight/weight) ratio. Since α-crystallin consists of two gene products namely αA- and αB-crystallins, we have tested the recombinant proteins on their ability to prevent thermal-stress induced AQP0 aggregation. In contrast to the general observation made with other target proteins, αA-crystallin exhibited better chaperone-like activity towards AQP0 compared to αB-crystallin. Neither post-translational modifications (glycation) nor C-terminus truncation of AQP0 have any appreciable effect on its thermal aggregation properties. α-Crystallin offers similar protection against thermal aggregation as in the case of the unmodified AQP0, suggesting that αcrystallin may bind to either intracellular loops or other residues of AQP0 that become exposed during thermal stress. Far-UV circular dichroism studies indicated a loss of αhelical structures when AQP0 was subjected to temperatures above 45°C, and the presence of α-crystallin stabilized these secondary structures. We report here, for the first time, that α-crystallin protects AQP0 from thermal aggregation. Since stress-induced structural perturbations of AQP0 may affect the integrity of the lens, presence of the molecular chaperone, α-crystallin (particularly αA-crystallin) in close proximity to the lens membrane is physiologically relevant.

## Introduction

Aquaporins (AQPs) are the family of homologous water and glycerol transporters expressed in eubacteria, archae, fungi, plants and animals [1]. In mammals, about 13 AQPs have been identified [2]. AQP0, formerly known as MIP (membrane intrinsic protein), is the first cloned member of this family of proteins [3]. It is expressed exclusively in elongated fiber cells [4]. AQP0 channels transport water [5]. Water flow is essential in the internal micro-circulatory system for the avascular lens [6]. AQP0 water permeability is regulated by pH and Ca^2+^/calmodulin [7]–[11]. Mutations and the C-terminus truncation of AQP0 are associated with development of cataract [12]–[14].

In the lens, AQP0 seems to have additional functions as well. Dunia *et al.,* [15] observed clustering of AQP0 reconstituted liposomes, suggesting AQP0 may be involved in cellular adhesion. Two-dimensional crystallization showed both single- and double-layered crystals [16]–[17], suggesting AQP0 could mediate cellular adhesion besides water transport. Furthermore, [18] showed that expression of AQP0 enhanced cell-to-cell adhesion in adhesion-dependent fibroblast cell cultures. Thus it appears that in the outer layers of the lens AQP0 can function as a water pore, whereas in the inner layers it is primarily involved in mediating adhesion between neighboring cells.

It is reported that AQP0 interacts with other lens crystallins in the following decreasing order: αA-, αB-, βB2- and γC-crystallins [19]. It was shown that α-crystallin, a multimeric protein (comprised of αA- and αB-crystallin subunits) belonging to the family of small heat-shock proteins, binds more readily to the lens membranes than other crystallins, and this binding was shown to increase with age and in cataracts [20]–[22]. Mulders *et al.,* [23] and Liang and Li [24] suggested that binding of α-crystallin to the membranes is mediated through AQP0. Amounts of αA-crystallin associated with the membranes were found to be higher than those of α-crystallin. It was also suggested that αB-crystallin may not be able to bind to the membranes in the absence of αA-crystallin [25]–[26]. However, Su *et al.,* [27] showed that αB-crystallin has a higher affinity to bind to membranes, and this binding can be enhanced by mild thermal stress [28]. The nature and significance of the binding of α-crystallin to the membranes is still not completely understood.

Α-Crystallin functions as a molecular chaperone towards other lens cytosolic proteins such as β- and γ-crystallins as well as other target proteins [29]–[31]. Increased aggregation of AQP0 was observed in transgenic mice expressing CRYAA N101D mutant of α-crystallin [32], indicating that the interaction between α-crystallin and AQP0 has some physiological relevance. However, whether α-crystallin functions as a molecular chaperone and prevents aggregation of AQP0 has not been addressed so far. In fact, there are no comprehensive studies on the interactions of α-crystallin with any membrane protein, or its functional significance. In long-lived tissues like the lens, proteins (including AQP0) are subjected to cumulative insults from metabolic (oxidants) and environmental (UV rays) stresses during the lifetime, which may affect protein conformation, leading to aggregation and loss of function. In this study, we have used OG solubilized AQP0 as a model to study temperature-mediated structural changes and aggregation, and the effect of α-crystallin and its constituent homooligomers, αA- and αB-crystallins.

## Materials and Methods

### Preparation of lens membranes

Snap-frozen calf eyes were obtained from a commercial supplier (Pelfrez, Little Rock, AK) and stored at –80°C until use. Decapsulated lenses were homogenized in ice-cold 10 mM phosphate buffered saline, pH 7.4 (PBS, from Sigma Chemical Co, St. Louis, MO). Soluble fraction, containing lens crystallins, was removed by centrifugation at 18,000 x g for 30 min. The pellet was washed 3 times with PBS, resuspended in PBS containing 7 M urea and stirred for 1 hr at 4°C. The urea-soluble fraction was separated by centrifugation at 18,000 x g for 30 min. The pellet containing lens membranes was washed twice with PBS-urea, twice with PBS and then resuspended in PBS. AQP0 was solubilized in 1% octyl β-D-glucopyranoside (octyl glucoside or OG). The detergent-solubilized protein was recovered by centrifugation (18,000 x g for 30 min).

### Thermal denaturation assays of AQP0

Calf lens AQP0 solubilized in 10% OG was diluted with 10 mM phosphate buffer, pH 7.4 to final OG concentration of 1%. In all of the assays, the final concentration of AQP0 was kept at 0.15 mg/ml. To initiate aggregation, the temperature of the AQP0 solution (0.15 mg/ml) was raised using a Julabo thermo-stated water bath connected to the Hitachi F-4000 fluorescence spectrophotometer. The extent of protein aggregation was measured as a function of time by monitoring the 90° scattering of 465 nm light. In some experiments, the aggregation was measured by measuring the absorbance at 360 nm on a Jasco J715 spectropolarimeter.

### Gel permeation chromatography profile of thermally stressed AQP0

Samples of OG- solubilized calf lens AQP0 were incubated at 50°C for various time intervals and resolved on gel permeation HPLC columns (SEC 3000 SW, 10 mm×600 mm, maintained at 50°C with the help of a water jacket). The mobile phase consisted of 10 mM PBS, 100 mM NaCl and 1% OG. The absorbance was monitored at 280 nm while the flow rate was maintained at 1 ml/min. In some experiments, bovine α–crystallin (1∶1 weight/weight ratio) was also added prior to the incubation of AQP0 at 50°C.

### Assays for measuring chaperone activity of α-crystallin against AQP0 and ζ-crystallin

In all chaperone assays, we have used either bovine α–crystallin (isolated from calf lens cortical fibers) or recombinant human α–crystallins (either homooligomers of αA-, or αB-crystallins or heterooligomers of αA- and αB-crystallins, at 3∶1 ratio). The chaperone to target protein concentration ratio was maintained at 1∶1 (weight/weight)) ratio, unless stated otherwise.

Thermal denaturation of calf lens AQP0 was performed, as described above, in the presence of α–crystallin. Aggregation of ζ-crystallin in 10 mM phosphate buffer, pH 7.4 containing 1% OG was initiated by increasing the sample temperature to 43°C. Similarly, aggregation of insulin in 10 mM phosphate buffer, pH 7.4, containing 1% OG was initiated by the DTT (dithiothreitol)-induced reduction of the disulfide bonds at 37°C. The extent of aggregation in both of these assays was measured either in the absence or in the presence of αA-, or αB-crystallin.

### Effect of glycation of AQP0 on the chaperone function of α-crystallin

In order to study the effect of glycation of AQP0 (a common post-translational modification in aging and in diabetes) on the chaperone function of α-crystallin, lens membranes were incubated without and with 1 M glucose for 4 days at 37°C as described earlier [33]. After the incubation, the membranes were washed with PBS, solubilized in 1% OG in PBS and then subjected to thermal stress in the absence or in the presence of non-glycated α-crystallin.

### Effect of C-terminus truncation of AQP0 on the chaperone function of α-crystallin

We have also tested the chaperone activity of α-crystallin in preventing the aggregation of C-terminus truncated AQP0. C-terminus truncated 22 kDa fragment of AQP0 was generated by limited proteolysis using Glu-C protease. Calf lens membranes were resuspended in 10 mM ammonium bicarbonate buffer, pH 4.5, and incubated at 37°C for 20 hrs at an enzyme to membrane protein ratio of 1∶20 (weight/weight). Following the incubation, the membranes were washed with cold PBS and solubilized in 1% OG in PBS. The resultant 22 kDa AQP0 fragment was then subjected to thermal stress in the absence or presence of non-glycated α-crystallin.

### Monitoring secondary structural changes of AQP0 during thermal-stress

Changes in the secondary structure of AQP0 during thermal stress were followed by far UV Circular dichroism (CD) measurements using a Jasco J-715 spectropolarimeter. Detergent-solubilized AQP0 (0.1 mg/ml in 1% OG, 10 mM phosphate buffer, pH∶7.4 containing 100 mM NaCl) was used in this assay. CD spectra, measured between 193–260 nm, were used to analyze the structural changes during thermal stress. In order to determine the chaperone function of α–crystallin towards AQP0 during thermal-denaturation, bovine α-crystallin was added in the assays. In these assays, the CD spectra were corrected by subtracting the background of a sample containing only α-crystallin in detergent and buffer.

### Gel electrophoresis

In order to characterize the protein aggregation, proteins were separated on a 12% SDS-polyacrylamide gel. The protein samples were incubated for 1 hour at 25°C in the SDS-PAGE sample buffer containing 50 mM Tris-HCl (pH 6.8), 2% SDS, and 0.1% β-mercaptoethanol before loading on the gel. After electrophoresis, the proteins were visualized by staining with Coomassie Brilliant Blue 250.

### Protein content

Protein content was estimated by the BCA protocol (kit from Pierce) using bovine serum albumin as the standard.

## Results and Discussion

### Thermal aggregation of AQP0 and its prevention by α-crystallin

**Figure 1:**
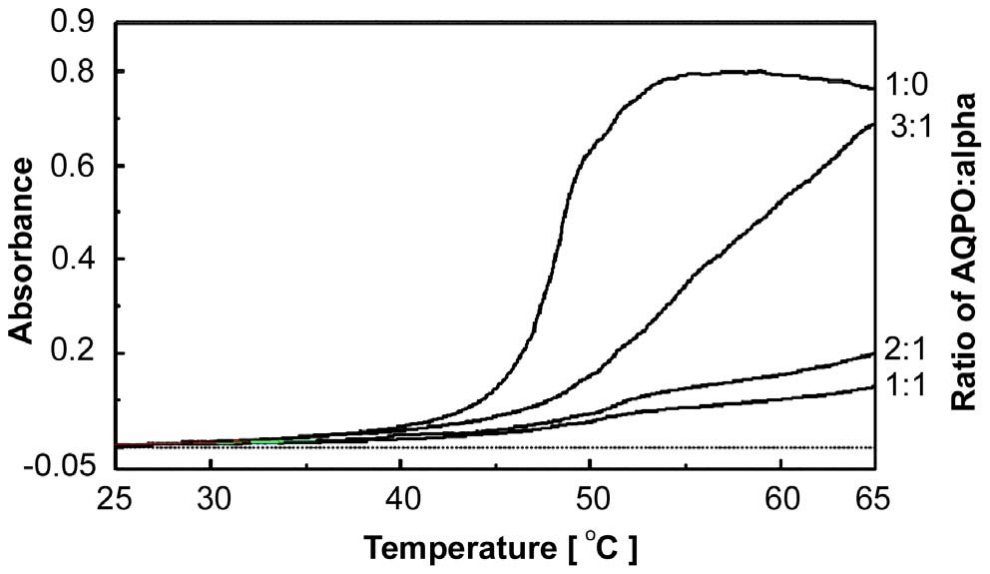
Thermal aggregation of AQP0 and protection by α-crystallin. Detergent solubilized AQP0 (0.15 mg/ml in 10 mM phosphate buffer, pH 7.4 containing 1% OG) was subjected to thermal stress by gradually increasing the temperature and the absorbance monitored at 360 nm on a Jasco 715 Spectropolarimeter.

**Figure 2:**
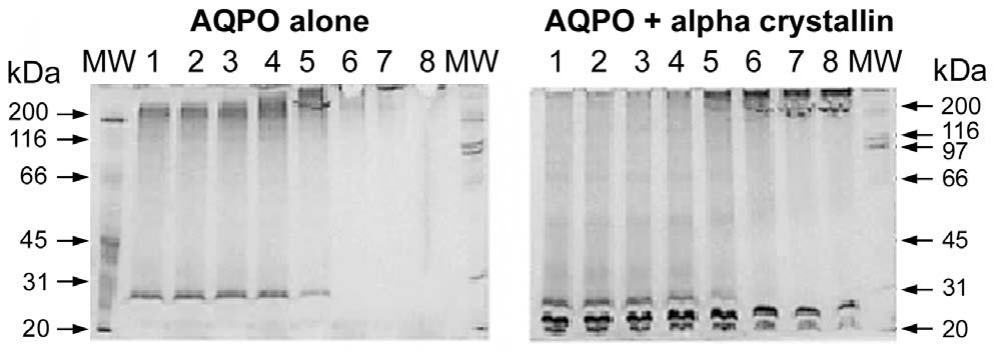
Gel electrophoresis analysis of thermal-denaturation of AQP0 and protection by α-crystallin. Detergent solubilized AQP0 (0.15 mg/ml in 10 mM phosphate buffer, pH 7.4 containing 1% OG) was subjected to thermal stress in a thermal cycler (5 min at each given temperature) without and with α-crystallin and resolved on 12% SDS-PAGE. Left panel - AQP0 alone and Right panel - AQP0 + α-crystallin. MW – molecular weight markers (range 20-200 kDa); lane 1 – 25°C; lane 2 – 30°C; lane 3 – 35°C; lane 4 – 40°C; lane 5 – 45°C; lane 6 – 50°C; lane 7 – 55°C and lane 8 – 60°C.

Although anecdotal evidence suggests that boiling lens membranes leads to aggregation of AQP0, this is the first systematic study to show it thermal-induced aggregation properties. Owing to its pivotal role in water transport and its involvement in cell-to-cell adhesion, understanding stress-induced aggregation of AQP0 is highly relevant in lens pathophysiology. Plasencia *et al.,* [34] have used thermal stress to study structural perturbations of spinach aquaporin (SoPIP2;1) in biomimetic systems. Our choice in using a similar technique resides in the convenience of manipulating conditions to induce structural perturbations in AQP0, and to demonstrate the chaperone function of α-crystallin. When OG solubilized AQP0 was subjected to thermal stress, we observed a gradual increase in the absorbance at 360 nm at temperatures above 40°C, indicating protein aggregation (Fig 1). Further, SDS-PAGE analysis showed a temperature-dependent loss of AQP0 monomers (26 kDa) with a concomitant increase in protein aggregates (>200 kDa) as shown in Fig 2 (left panel, lanes 5–8). Although the pattern of AQP0 aggregation is similar to that observed with spinach AQP (SoPIP2;1)[34], the threshold to tolerate thermal-stress seems to be about 10°C lower (40°C for AQP0 vs 50°C for SoPIP2;1). It is interesting to note that thermal stress-induced high molecular weight aggregates of AQP0 did not enter the SDS-PAGE gels (Fig.2). Earlier studies have shown that aqua-glyceroporins (glycerol facilitator), archaeal aquaporin (AqoM) and spinach aquaporin (SoPIP2;1) also form large aggregates which do not enter the SDS-PAGE gels [34]–[36]. It appears that aquaporins form SDS-resistant aggregates similar to other membrane proteins including the porins as reported earlier [37. 38].

α-Crystallin is known to prevent the aggregation of several target proteins induced by UV-radiation, DTT and temperature [31], [39]. In order to find out whether α-crystallin exhibits similar chaperone-like activity for AQP0, we have used different concentration ratios of AQP0 to bovine α-crystallin (1∶1; 2∶1; 3∶1, respectively) in the chaperone assays. α-Crystallin prevented the thermal aggregation of AQP0 in a concentration-dependent manner and nearly eliminated aggregation at the ratio of 1∶1 (Fig 1). SDS-PAGE analysis showed a progressive loss of monomeric subunits of AQP0 as well as α-crystallin above 45°C with a concomitant increase in the formation of AQP0-α crystallin complexes as indicated by the appearance of bands at ∼200 kDa (Fig 2, right panel, lanes 5–8).

### Gel permeation chromatography profile of thermally stressed AQP0 and prevention of aggregation by α–crystallin

**Figure 3:**
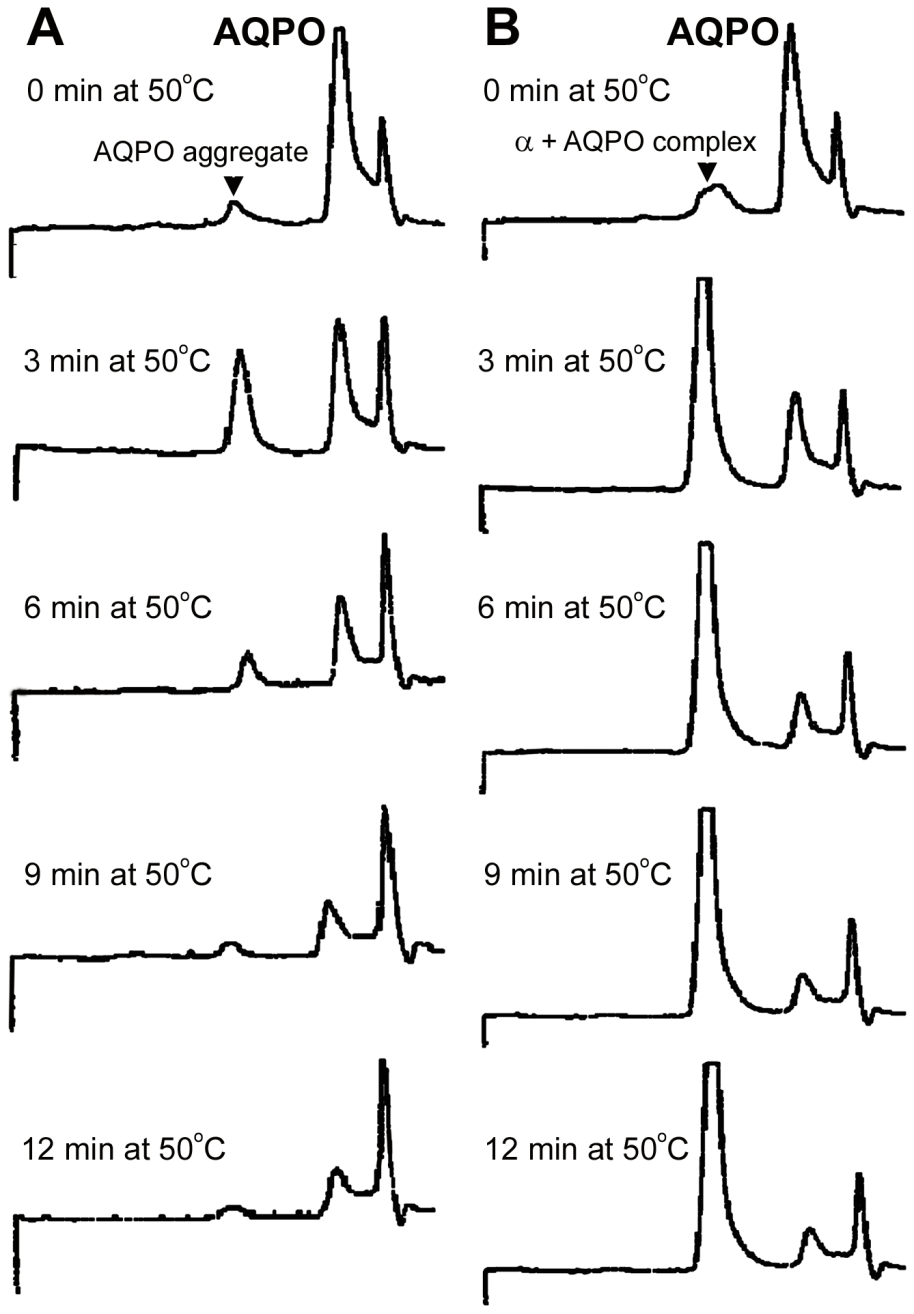
HPLC temporal profile of thermally stressed AQP0. A: Samples of OG solubilized calf AQP0 (0.15 mg/ml in phosphate buffer, pH 7.4 containing 100 mM NaCl and 1% OG) were incubated at 50°C for various time intervals. Note the AQP0 monomer resolved as a major peak at 26 kDa protein and the AQP0 aggregate at ∼ 1000 kDa (see 3 min at 50°C). When the AQP0 was maintained at 50°C for 6 min or more, these AQP0 aggregates also disappeared. B: Addition of α-crystallin in the incubation mixture resulted in the formation of α-crystallin and AQP0 complexes resolved at ∼800 kDa.

Thermal stress initially resulted in unfolding of AQP0 leading to the formation of soluble aggregates at ∼ 1000 kDa with a concomitant loss of monomers at 26 kDa (Fig 3; 3 min). However, prolonged thermal stress resulted in the formation of larger aggregates which are insoluble and failed to elute through the columns (Fig 3; 6–12 min). Presence of α-crystallin seems to have favored the formation of the α-crystallin/AQP0 complex which resolved at ∼ 800 kDa. These gel permeation chromatography results are consistent with the results obtained from light scattering and gel electrophoresis (Figs 1 and 2). The results obtained from SDS-PAGE (Fig2 left panel, lanes 6–8) suggest that AQP0 monomers completely disappear at 50°C and beyond, leading to the formation of large aggregates >200 kDa. Fig 2 (right panel, lanes 6–8) suggests that α-crystallin interacts with AQP0 and the resultant AQP0-α-crystallin complex yields bands at ∼ 200 kDa at 50°C and beyond; however, part of the AQP0-α-crystallin complex did not enter the gel (Fig 2 right panel, lanes 6–8), suggesting the formation of high molecular weight aggregates (> 200 kDa). On the other hand, on the gel-filtration column this band was resolved at 800 kDa.

### Specificity in the α-crystallin gene products towards AQP0

**Figure 4:**
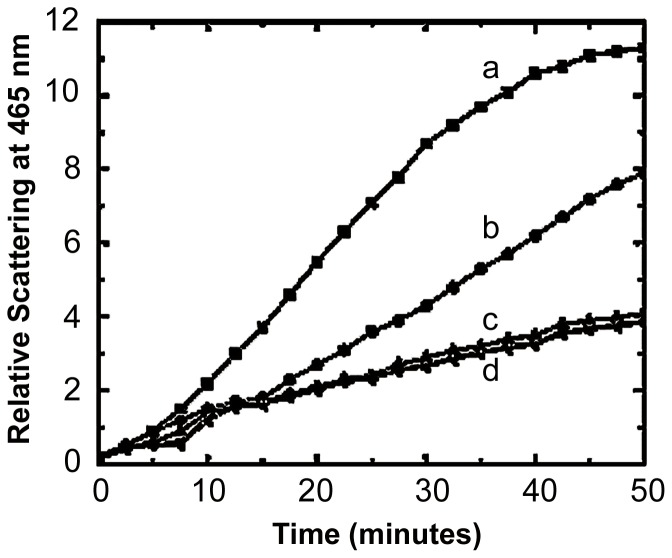
Chaperone-like activity of reconstituted human α-crystallin and its gene-products. Thermal aggregation of (a) AQP0 (0.15 mg/ml) at 49°C, (b) in presence of recombinant human αB- crystalline (0.15 mg/ml) (homooligomer), (c) recombinant human αA-crystallin (0.15 mg/ml) (homooligomer), and (d) in presence of recombinant human α-crystallin (0.15 mg/ml) (heterooligomer of αA- and αB-crystallins at 3∶1 ratio).

α–Crystallin is comprised of two gene products, αA- and αB-crystallin. It is well-known that αB-crystallin is ubiquitously expressed in many non-lenticular cells, whereas αA-crystallin is found only in trace quantities in non-lenticular tissues [40]. This difference in tissue- specific expression suggests that these gene products might have evolved to play distinct physiological functions. In order to find out if these two gene products exhibit any specificity in preventing aggregation of AQP0, we have used human recombinant α–heterooligomer (prepared by mixing recombinant human αA- and αB-crystallins in a ratio of 3∶1 weight/weight) as well as human recombinant αA- and αB-homooligomers, which have been well-studied in our laboratory with other target proteins. We have also compared the chaperone activity of bovine α–crystallin with that of recombinant human α-crystallin. The results showed no noticeable variation (data not shown). It has been generally observed that αB-crystallin is a more effective chaperone than αA-crystallin [29]. Our observation suggests that αA-crystallin is more effective in preventing thermal-aggregation of the membrane protein, AQP0 (Fig 4). The existence of αA-crystallin and AQP0 in close proximity in the lens would have a physiological significance. This is supported by the work of [41] who showed that αA-crystallin knockouts exhibited irregular localization of AQP0 in the lens membranes, suggesting that normal levels of αA-crystallin are essential to maintain normal membrane architecture.

### Evidence that the detergent has no effect on the thermal stress or chaperone function of α-crystallin

**Figure 5:**
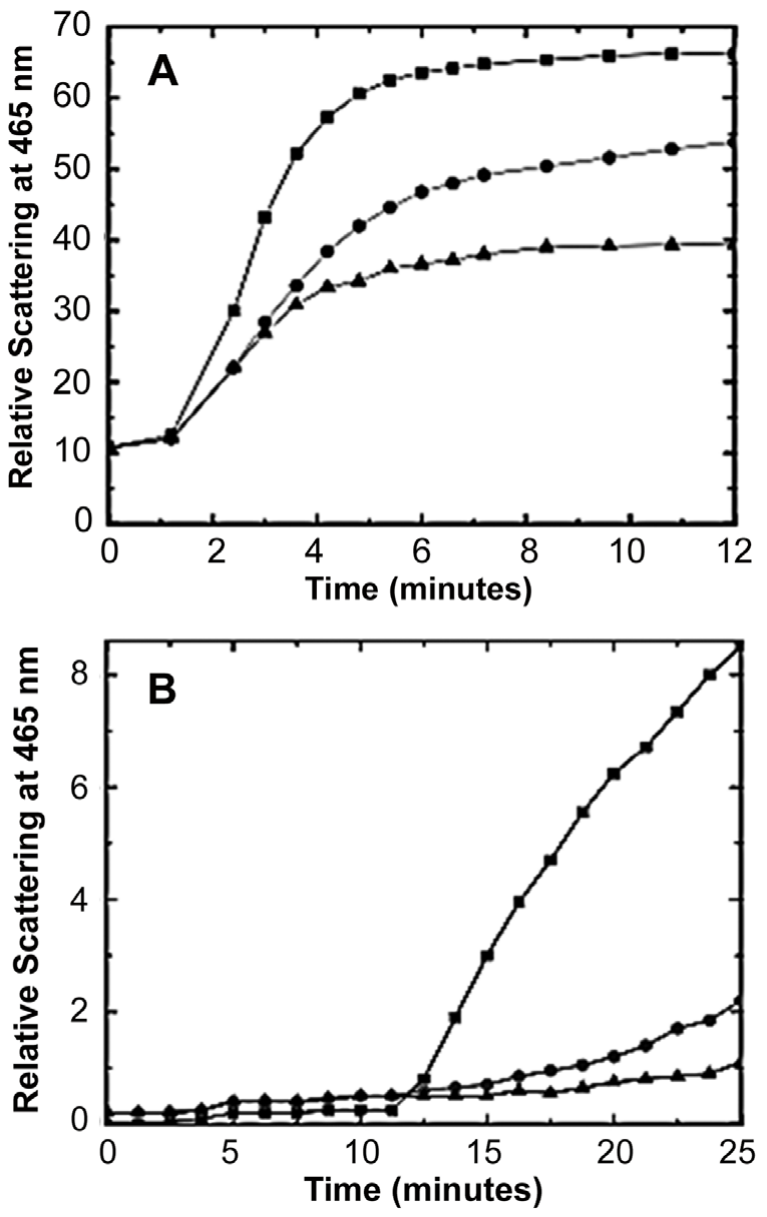
Chaperone-like activity of human α-crystallin gene products against target proteins. **A**: (•) Recombinant human αA-crystallin (0.1 mg/ml) (homooligomer) and (▴) recombinant human αB-crystallin (0.1 mg/ml) (homooligomer), against (▪) thermal aggregation of ξ-crystallin (0.1 mg/ml) at 43°C. **B**: (•) Recombinant human αA-crystallin (0.2 mg/ml) (homooligomer) and (▴) recombinant human αB-crystallin (0.2 mg/ml) (homooligomer), against (▪) DTT-induced aggregation of insulin (0.2 mg/ml) at 43°C.

Generally, all the chaperone assays are carried out in buffers under low ionic strength and salt conditions. Under these conditions we have shown earlier that the recombinant αB-crystallin offers much better protection than αA-crystallin against either DTT-induced aggregation of insulin or thermally-induced aggregation of ξ-crystallin [39]. In our present study with AQP0, we have used octyl glucoside as the detergent to keep it in a soluble form. This raises a possibility that the structural features of αA- and αB-crystallin might have been altered in the micelle environment of OG. To test this possibility, we have carried out the chaperone assays of α-crystallin against thermally-induced aggregation of ξ-crystallin and DTT-induced aggregation of insulin under conditions used for AQP0 chaperone assays. The time course for the aggregation of thermally stressed ξ-crystallin (at 43°C) or of the DTT-reduced insulin is essentially the same in the presence of OG (Fig. 5A) or in its absence. Further, OG did not alter the chaperone specificity of αB- over that of αA-crystallin for ξ-crystallin or insulin (Fig 5B). These results indicate that OG is not responsible for the preferential interaction of αA-crystallin (hence increased chaperone-like activity) with AQP0, compared to αB-crystallin.

### Effect of C-terminus modifications of AQP0 on its thermal aggregation and the binding of α–crystallin

**Figure 6:**
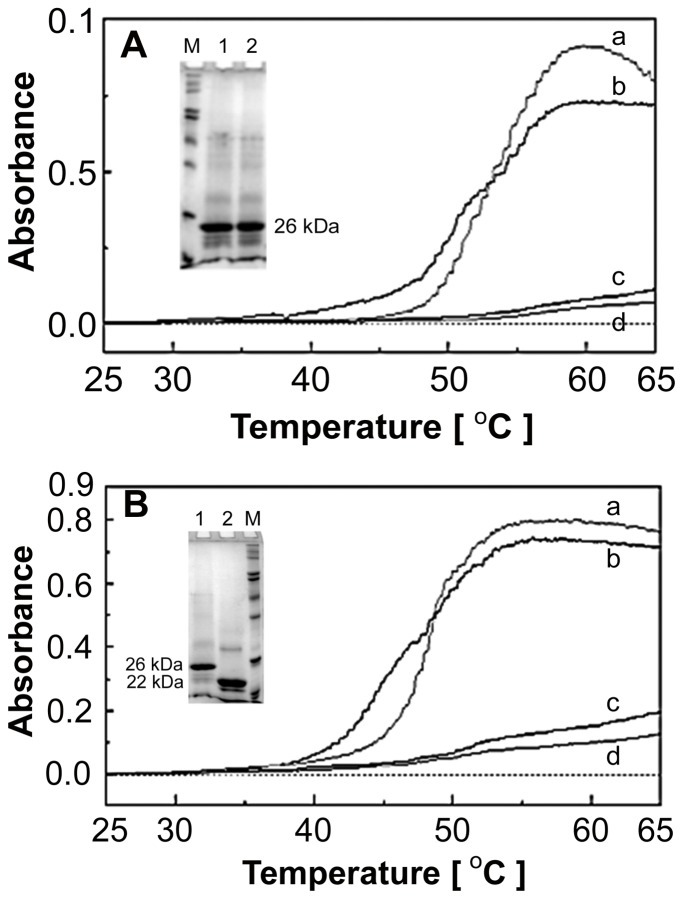
Effect of post-translational modifications and C-terminus truncation of AQP0 on thermal aggregation and chaperone function of α–crystallin. A: Effect of glycation of AQP0 on thermal aggregation and chaperone function. Lens membranes were incubated with 1 M glucose for 4 days. Glycated AQP0 was solubilized in 1% octyl glucoside and subjected to thermal stress. a - glycated AQP0; b - non-glycated AQP0; c - glycated AQP0 + α–crystallin; d - non-glycated AQP0 + α–crystallin. Inset: SDS-PAGE of *in vitro* glycated AQP0. M – Markers; 1 – Control incubation; 2 - 1 M glucose incubation. B*:* Effect of C-terminus truncation of AQP0 on thermal aggregation and chaperone function: Lens membranes were treated with Glu-C protease and the resultant C-terminus truncated AQP0 was solubilized in 1% octyl glucoside, and then subjected to thermal stress. a - control AQP0; b - C-terminus truncated AQP0; c - control AQP0 + α-crystallin; d - C-terminus truncated AQP0 + α-crystallin. Inset: SDS-PAGE of C-terminus truncated AQP0. 1 – Control AQP0; 2 – Glu-C protease digested AQP0; M – Markers.

Earlier studies have shown that the cytoplasmic C-terminus region of AQP0 has consensus sites for post-translational modification such as glycation (K228, K238, K251) [42], phosphorylation (S235) [43] as well as calmodulin-binding (225-241) [[7]. Further, Ball *et al*., [44] showed age- and cataract-dependent truncations of the C-terminus of AQP0. In order to understand the effect of these modifications on the thermal aggregation of AQP0 and its interaction with α-crystallin, AQP0 was glycated *in vitro* as described earlier [42] and used in the thermal aggregation assays in the presence and in the absence of α–crystallin. Using mass spectrometry, we have shown earlier a mass shift equivalent to one, two and three glucose adducts in AQP0 samples incubated with 1 M glucose [42]. As shown in Fig 6A, the glycation of AQP0 has no effect on thermal aggregation or on the chaperone function of α-crystallin.

In order to study the effect of C-terminus truncation of AQP0, we have used Glu-C protease to cleave the C-terminus region and used the resultant 22 kDa AQP0 in the thermal aggregation assays in the presence and in the absence of α-crystallin. As shown in Fig 6B, truncation of the C-terminus region has no effect on the thermal aggregation of AQP0 or the chaperone function of α-crystallin. These studies reveal that the C-terminus is not the binding site for α-crystallin. This implies that the B and/or the D intracellular loops of AQP0 or some of the membrane-embedded residues that may become exposed under thermal stress are the possible binding sites for α-crystallin.

### Thermally induced secondary structural changes of AQP0 and its prevention by α- crystalline

**Figure 7:**
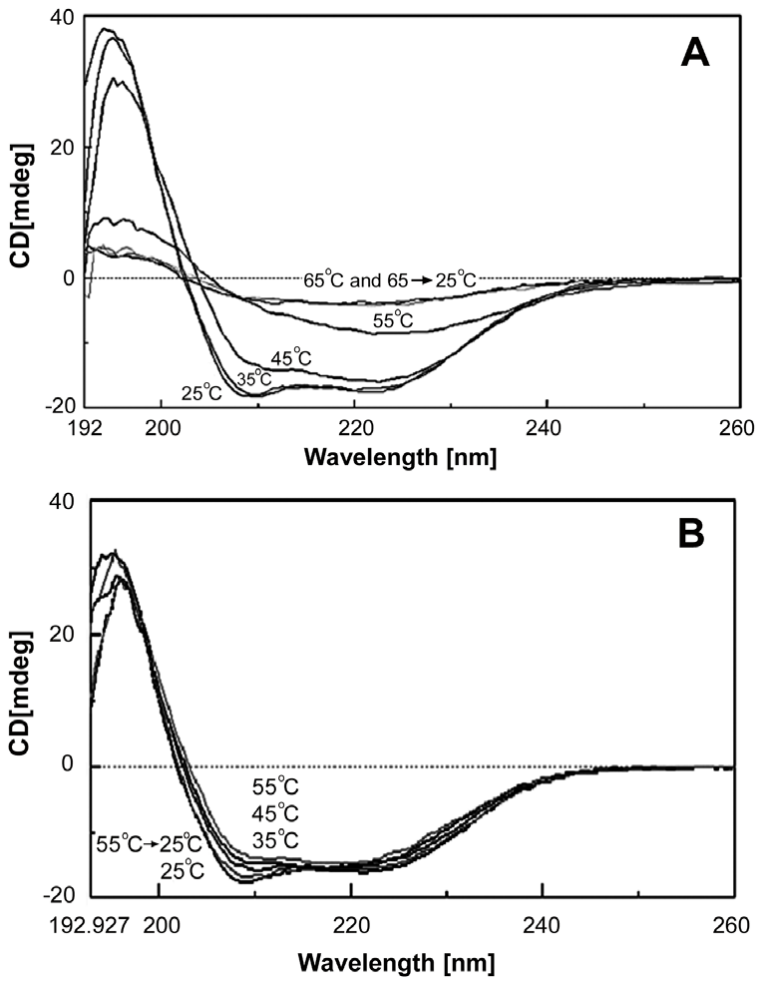
Secondary structural changes of AQP0 during thermal stress and protection by α–crystallin. A*:* Detergent solubilized AQP0 (0.1 mg/ml in 10 mM phosphate buffer, pH 7.4 containing 100 mM NaCl and 1% OG) was subjected to thermal stress for 5 min at a given temperature. Following the thermal stress, the protein solution was immediately cooled to 25°C and Far UV CD studies were done at 25°C using a circular dichroism spectropolarimeter. Note progressive loss of α-helical structures with increasing temperature and these changes are irreversible. B*:* The same assay was done in the presence of bovine α–crystallin (at a ratio of 1∶1 weight/weight).

We have investigated the effect of thermal stress on the secondary structures of detergent solubilized AQP0 by measuring its far-UV CD spectra as a function of temperature from 25°C to 65°C (Fig. 7A). Deconvolution of the spectra [45] shows that the α–helical content of AQP0 is about 50% when the temperature is below 40°C, which is in reasonable agreement with crystallographic data [46]. A small decrease in the native secondary structure was observed at 45°C; at about 55°C there is a significant loss of secondary structure with some residual structure. At higher temperatures even this residual structure was completely lost (Fig. 7A). These results are in agreement with the thermally induced changes observed in secondary structures measured for OG solubilized spinach AQP0 (SoPIP2;1) [34]. Sagne *et al* [47] have proposed that proteins susceptible to SDS-resistant aggregation have detectable secondary structure in the presence of SDS. Conditions such as thermal stress cause partial unfolding of the residual structure and trigger aggregation. Our CD studies show that there is detectable residual secondary structure between 45°C and 55°C. It is likely that these partially unfolded residual structures undergo aggregation, which is resistant to SDS (Fig. 1, left panel). Some of the membrane proteins are known to revert to their native secondary structure over a limited temperature range. Above this range, the interactions required to keep the protein and detergent in the soluble form are disrupted, leading to the oligomerization and aggregation of the protein [48]. Whether such reversal of the secondary structure occurs in the case of AQP0 has been investigated by us using far-UV CD spectroscopy and the results are shown as supplemental data (Fig. S1). Fig. S1, panel A suggests that the CD spectrum of the sample recorded at 25°C overlaps with the spectra of the samples that were heated to 40°C and brought back to 25°C. Similar phenomenon was exhibited by AQP0 at 42.5°C as well, as shown in the Fig. S1, panel B. However, at temperatures higher than 42.5°C, secondary structural changes as observed by CD are irreversible (Fig. S1, panels C and D). Presence of α–crystallin had a marked effect in preventing thermally induced secondary structural changes of AQP0. As shown in Fig. 7B, the CD spectra obtained at all temperatures tested (25° C – 55 °C) retained a significantly greater proportion of α–helical structure, which correlates with the minimization of light scattering shown above (Fig 1).

Loss of secondary structures under thermal stress and protection by α–crystallin has profound physiological significance. In the lens, significant amounts of α–crystallin have been shown to be associated with lens membranes with age and cataracts [23]. Liang and Li [24] suggested that α–crystallin binding to the membranes is mediated through AQP0. Further, Liu and Liang [19] demonstrated that the extent of binding interactions decrease in the following order - αA-, αB-, βB2- and γC-crystallin. Stress-induced structural perturbations of AQP0, the major membrane protein, may result in protein aggregation and loss of function leading to lens opacification. Presence of αA-crystallin in close proximity to AQP0 would be physiologically significant. This study, for the first time, brings to light the chaperone function of α-crystallin against membrane proteins such as AQP0, which is involved in maintaining lens fiber cell homeostasis.

## Supporting Information

Figure S1 **Temperature-dependent secondary structural changes of AQP0 studied by far-UV CD:** The spectra of detergent solubilized AQP0 (0.1 mg/ml in 10 mM phosphate buffer, pH 7.4 containing 100 mM NaCl and 1% OG) was recorded at 25°C. Samples of AQP0 were heated to 40°C (panel A), 42.5°C (panel B), 45°C (panel C) or 50°C, respectively and far-UV spectra recorded. Each of the protein samples was cooled back to 25°C and the spectra recorded again under the same conditions.
(TIF)
