# Supplementary figures and images for: Thermal Stress Induced Aggregation of Aquaporin 0 (AQP0) and Protection by α-Crystallin via Its Chaperone Function
Source: PLoS One. 2013 Nov 27;8(11):e80404. doi: 10.1371/journal.pone.0080404 (PMC3842347; doi:10.1371/journal.pone.0080404)

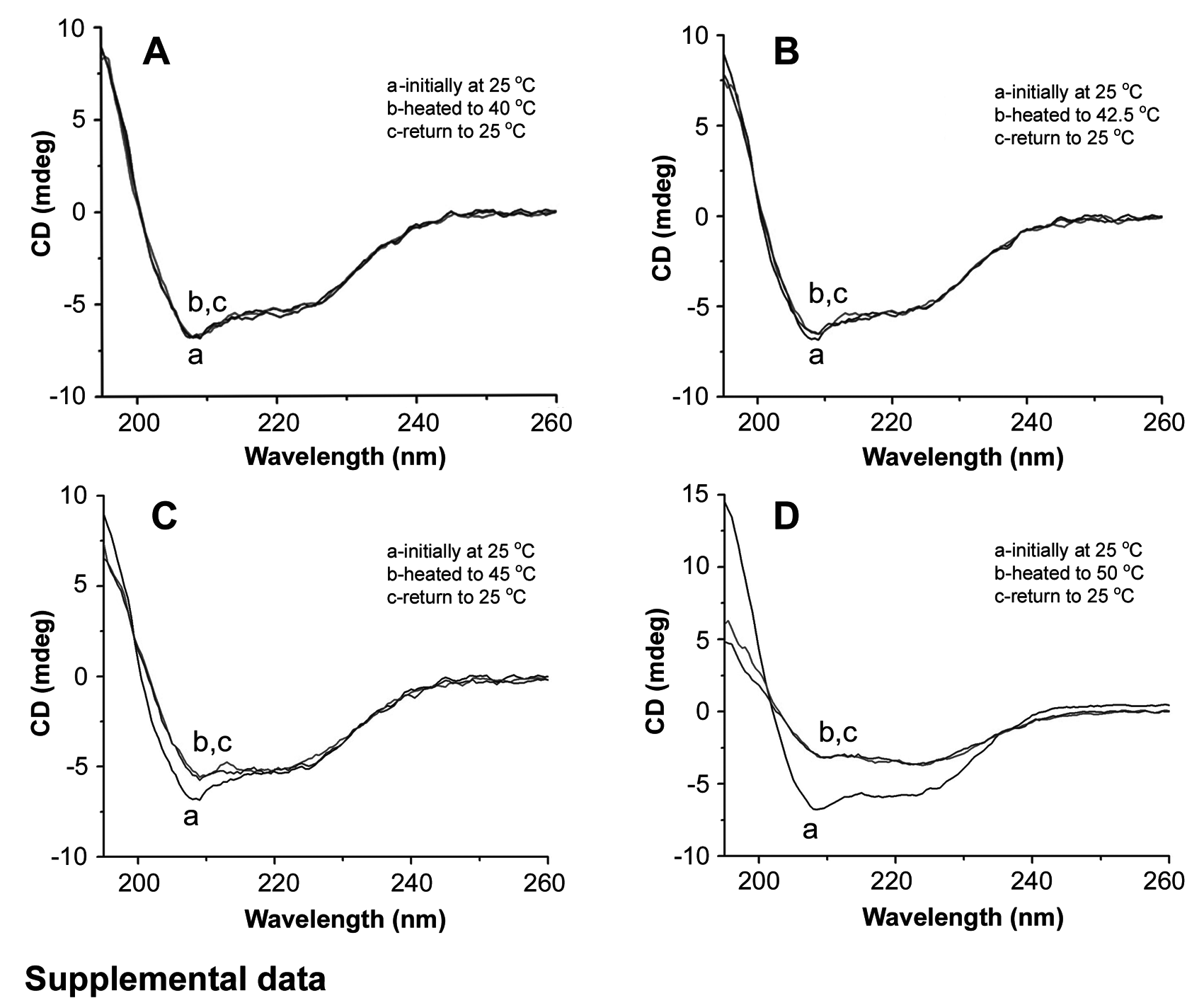

Supplement: Figure S1 — Temperature-dependent secondary structural changes of AQP0 studied by far-UV CD: The spectra of detergent solubilized AQP0 (0.1 mg/ml in 10 mM phosphate buffer, pH 7.4 containing 100 mM NaCl and 1% OG) was recorded at 25°C. Samples of AQP0 were heated to 40°C (panel A), 42.5°C (panel B), 45°C (panel C) or 50°C, respectively and far-UV spectra recorded. Each of the protein samples was cooled back to 25°C and the spectra recorded again under the same conditions. (TIF) [file pone.0080404.s001.tif]
